# Cuproptosis: emerging biomarkers and potential therapeutics in cancers

**DOI:** 10.3389/fonc.2023.1288504

**Published:** 2023-11-07

**Authors:** Min Wang, Lianwen Zheng, Shuai Ma, Ruixin Lin, Jiahui Li, Shuli Yang

**Affiliations:** ^1^ Department of Obstetrics and Gynecology, The Second Hospital of Jilin University, Changchun, China; ^2^ Department of Hepato-Biliary-Pancreatic Surgery, The Second Hospital of Jilin University, Changchun, China

**Keywords:** copper, cuproptosis, cell death, cancer, metabolism, immunotherapy, molecular targeted therapy

## Abstract

The sustenance of human life activities depends on copper, which also serves as a crucial factor for vital enzymes. Under typical circumstances, active homeostatic mechanisms keep the intracellular copper ion concentration low. Excess copper ions cause excessive cellular respiration, which causes cytotoxicity and cell death as levels steadily rise above a threshold. It is a novel cell death that depends on mitochondrial respiration, copper ions, and regulation. Cuproptosis is now understood to play a role in several pathogenic processes, including inflammation, oxidative stress, and apoptosis. Copper death is a type of regulatory cell death(RCD).Numerous diseases are correlated with the development of copper homeostasis imbalances. One of the most popular areas of study in the field of cancer is cuproptosis. It has been discovered that cancer angiogenesis, proliferation, growth, and metastasis are all correlated with accumulation of copper ions. Copper ion concentrations can serve as a crucial marker for cancer development. In order to serve as a reference for clinical research on the product, diagnosis, and treatment of cancer, this paper covers the function of copper ion homeostasis imbalance in malignant cancers and related molecular pathways.

## 1 Introduction

**Figure 1:**
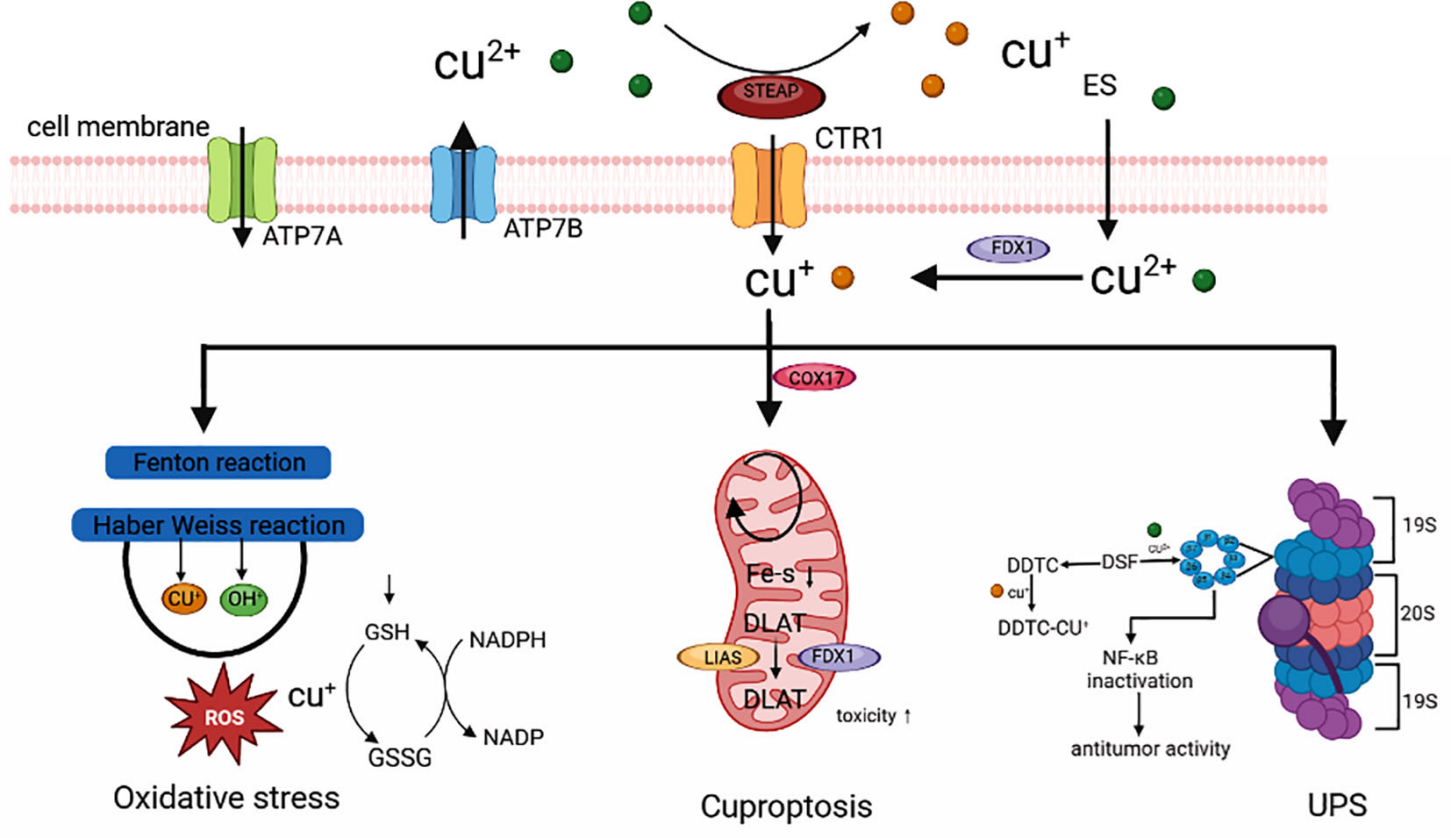
Cuproptosis is involved in the occurrence and development of cancer (3).

Cell death plays an important role in maintaining normal body homeostasis by inhibiting the uncontrolled proliferation of tumor cells and other biological processes. Cell death includes RCD and non-RCD (1). RCD is a genetically determined cell-active programmed death, including apoptosis, iron death, pyroptosis, necroptosis, etc., which can be induced by inducing the generation of Reactive Oxygen Species (ROS), regulating protein ubiquitylation, acetylation, and other functionally regulating cell death (2) (**Table 1**). Tsvetkov et al. reported a new mechanism of cell death different from the known ones and named it cuproptosis. Cuproptosis is closely related to cellular mitochondrial respiration: excess intracellular copper can be transported to the mitochondria via ion carriers and bind directly to the lipoylation component of the mitochondrial respiratory tricarboxylic acid cycle (TCA). Copper ions can interfere with iron-sulfur clusters, which in turn causes lipoylation protein aggregation and iron-sulfur cluster protein loss, inducing proteotoxic stress and ultimately cell death (3, 4). Previous findings have shown that, compared with normal human serum and tissues, copper ions are found at higher levels in the serum and tumor tissues of patients with a variety of human malignant tumors (5). Cuproptosis further reinforces the importance of cell death in tumorigenesis and progression and reveals the mechanisms of malignant tumorigenesis and progression, which provides a more theoretical basis for the search for new therapeutic strategies (6) (**Figure 1**).

**Table 1:** Cell death pathways and their characteristics.

Cell death pathway	Morphological changes	Biochemical characteristic
Cuproptosis	Cell membrane rupture, fatty acylated protein aggregation, Fe-S reduction.	Binding of copper ions to lipoylated modified proteins.
Ferroptosis	Rupture of the outer mitochondrial membrane, reduction of mitochondrial ridges, and mitochondrial membrane density increased, normal nucleus.	Iron ion and ROS accumulation, system Xc - activation, GSH consumption, lipids Peroxide.
Autophagy	Formation of double membrane autolysosomes, including large autophagy, microautophagy and partner-mediated autophagy.	LC3 -Ito LC3 -II conversion and substrate degradation.
Pyroptosis	Organelle loss, cell membrane rupture, DNA condensation rupture, release Radioactive pro-inflammatory cytokines.	Activation of caspase and gastrin, the release of neutrophil elastase and myeloperoxidase by a large number of proinflammatory factors, and activation of PAD4.
Necrosis	Plasma membrane rupture, cytoplasmic organelle swelling, chromatin concentration.	ATP depletion, protein hydrolysis and DAMP release involving calpain and cathepsin.
Apoptosis	Agglutination of chromatin, formation of apoptotic bodies, disintegration of the cytoskeleton, reduction of cell and nuclear volume.	Caspase activation, PS exposure, mitochondrial membrane potential.

ROS, reactive oxygen species; GSH, Glutathione; LC3,Microtubule-associated protein light chain3; DNA, deoxyribonucleic acid; PAD4,Peptidylarginine deiminase 4; ATP, Adenosine Triphosphate; DAMP, Damage-associated molecular patterns; PS, Phosphatidyl serine.

## 2 Copper

One of the most essential heavy metals in the human body is copper (7). Copper is also a significant cofactor in biological redox reactions and is involved in various biosynthetic processes in the body (8). Food is the primary source of copper intake, and both Cu+ and Cu2+ forms of copper can be found in the human body. In the human body, the copper ion is present as Cu2+, which is then converted to Cu+ by reductase after binding to the divalent metal transporter 1 (DMT1), which then enters the cell after binding to the transmembrane copper transporter 1 (CTR1) (9, 10). The Cu+ bound copper proteins enter the bloodstream through particular organelles and are then transported to the tissues and organs where they are required (11). A sufficient amount of copper in cells is essential for life activities as a catalytic cofactor for enzymes involved in energy conversion, iron collection, oxygen transport, and intracellular oxidative metabolism (12). Copper levels in the human body are in dynamic balance under physiological settings. If copper homeostasis is disturbed in an organism, some issues are brought about, including the induction of cell death and the suppression of angiogenesis (13, 14). Iron intake, antioxidant activity, and mitochondrial respiration are all highly dependent on copper (15). Copper plays a crucial role in cell signaling, a biological process that regulates the environment inside the body’s cells (16).

## 3 Homeostatic regulation of copper

The concentration of copper in normal cells is very low. It mainly prevents the accumulation of harmful intracellular free copper through the Dynamic equilibrium mechanism across concentration gradients, thus maintaining the homeostasis of copper in cells (17). In eukaryotic cells, mitochondria are important organelles for copper storage and mobilization; Cytochrome C oxidase copper chaperone protein 17 located in the gap between the cytoplasm and mitochondrial membrane can bind Cu+, carry Cu+ from the cytoplasm to the mitochondrial gap, and transmit it to cytochrome oxidase deficient homolog 1, transfer to the cytochrome C oxidase II and I subunits to activate enzyme activity in the respiratory chain. Regulating the expression of copper Ion channel proteins can affect the transport and metabolism of copper ions (18). Copper signals can be inhibited by copper-selective chelating agents, activated by metal ion carriers to increase copper levels, or redistributed spatially and temporally to cells and subcells (19). Ceruloplasmin (CP) is the main protein carrier for exchangeable copper in mammalian plasma, with the functions of copper translocation and metal chaperone, jointly maintaining appropriate intracellular copper bioavailability and ensuring the metallization of copper-dependent enzymes (20, 21). CP is a multicopper oxidase that initially binds to most of the copper output from liver cells to the systemic circulation, and enters the secretion pathway through copper transport driven by ATP7B. Due to the rapid degradation of ceruloplasmin when not bound to metals, the concentration of ceruloplasmin in plasma can serve as a biomarker for copper deficiency in the body. Considered the primary transporter of copper ions entering cells, solute carrier family 31 member 1 (SLC31A1) is a member of the CTR family with an extracellular copper binding domain. It is a high-affinity transporter protein for reduced Cu+ and has high expression levels in human cancer tissues (22). However, copper’s natural redox properties make it advantageous and potentially harmful to cells (23). Low copper ion concentrations have been linked to albinism, osteoporosis, and other illnesses (24). Because copper is also found in the brain’s copper protein in the central nervous system, a copper deficiency can impact brain development, demonstrating copper’s vital role in the body (25, 26).

## 4 Cancer prevention and control

Cancer is a major global public health problem, which seriously threatens human health, and incidence rate and mortality are rising. The International Agency for Research on Cancer (IARC) has released a 2020 global cancer statistics report, with approximately 19.3 million new cancer cases worldwide and nearly 10 million deaths from cancer. Female breast cancer has surpassed lung cancer to become the cancer with the highest incidence rate, accounting for 11.7% of all new cancer cases, followed by lung cancer (11.4%), rectal cancer (10.0%), prostate cancer (7.3%) and gastric cancer (5.6%). Lung cancer is the leading cause of death among all cancers, accounting for 18% of all cancer deaths. The second were rectal cancer (9.4%), liver cancer (8.3%), gastric cancer (7.7%) and female breast cancer (6.9%). Compared to 2018, there has been an increase in data, resulting in huge population losses and a heavy economic and medical burden. As the population ages, this trend will become more apparent. It is expected that there will be 28.4 million new cases of cancer in 2040, an increase of 47% compared to 2020. This result suggests that promoting cancer prevention and control measures and conducting early diagnosis and treatment of cancer are crucial for global cancer prevention and control.

## 5 Correlation between copper and cancers

Direct copper binding to the TCA’s lipoylation component causes proteotoxic stress, which ultimately results in cell death (27). Abnormal accumulation of copper ions can facilitate malignant cell transformation (28). The serum or cancer tissue of patients with various malignancies has been found to contain high copper contents (29). An imbalance in copper alters lipid metabolism, glycolysis, and insulin resistance while affecting mitochondrial respiration. Unbalanced levels of copper activate vascular endothelial growth factor (VEGF), fibroblast Growth Factor2 (FGF2), tumor necrosis factor (TNF), and interleukin (IL) -1, which start ULK1 and ULK2, which regulate autophagy. This promotes angiogenesis, enabling cancer cell proliferation and spread (30). Human angiogenesis is the result of the combined action of multiple small molecules such as VEGF, FGF2, IL-1, IL-6, and IL-8. Research has shown that copper can stimulate angiogenesis by directly binding to angiopoietin or by binding to HIF-1 to activate these small molecules. Abnormally elevated copper within cells can activate autophagy related ULK1 and ULK1 dependent signaling pathways to induce autophagy, thereby inhibiting copper induced cell apoptosis. Both copper ion carriers and copper chelators are regarded as therapeutically beneficial treatment agents for conditions linked to copper homeostasis and are often used. Using biological markers in copper ion carrier clinical trials will be crucial for creating copper-targeted treatment approaches. The Ubiquitin Proteasome System (UPS), copper deficiency-induced angiogenesis inhibition in cancer cells, and cuproptosis, as identified by recent studies, are the primary mechanisms by which copper has been shown to cause cancer cell death (31).

### 5.1 Catalyzing oxidative stress

The death of normal or cancer cells results from oxidative stress caused by altered oxidative-antioxidant equilibrium *in vivo* and is primarily characterized by high ROS concentrations (32, 33). The Cu+ in the body maintains the overall copper homeostasis through the absorption and excretion of copper. Excessive copper drives the Fenton reaction, producing a large amount of ROS, causing protein oxidation, DNA damage, nuclear damage, and dysfunction of mitochondria and various enzymes. Oxidative stress caused by copper primarily manifests itself in two ways. ROS can directly oxidize and cleave some copper complexes through the Fenton reaction, leading to necrotic apoptosis and toxic damage to cancer cells (34). Copper also mediates the synthesis of the most active - OH, which causes elevated ROS concentrations in cancer cells and kills cancer cells. Copper can deplete the antioxidant glutathione (GSH) by oxidizing reduced GSH to oxidized glutathione disulfide (GSSG), which disrupts the GSH-related antioxidant defence system and reduces the system’s ability to scavenge highly reactive -OH, which causes cancer cells to die (35). Copper complexes can induce apoptosis and autophagy through mitochondrial malfunction caused by oxidative stress because copper-induced high quantities of ROS are likewise fatal to mitochondria (36, 37). According to studies, the copper chelator elesclomol (ES), a highly lipophilic copper-binding molecule that chelates extracellular Cu2+, forms an ES-Cu2+ complex that transports copper to mitochondria for redox reactions and causes oxidative stress, which causes cancer cells to undergo apoptosis (38).

### 5.2 UPS

One of the mechanisms for the breakdown of many proteins in the human body is the UPS (39). UPS is essential in cancer cell growth, apoptosis, angiogenesis, and metastasis. Cu2+ can inhibit the proteasome through direct binding, according to studies (40). Disulfiram (DSF) (41) and other copper complexes have been employed as proteasome inhibitors in cancer treatment. A promising anticancer drug, DSF is an acetaldehyde dehydrogenase inhibitor that binds to Cu2+ to kill cancer cells (42). Diethyldithiocarbomate (DDTC) is similarly quickly formed *in vivo* from DSF (43, 44). It has been discovered that DDTC can form a dinuclear complex with Cu+, or DDTC-Cu+ and that this copper complex causes an accumulation of ubiquitinated proteins, an increase in p27, and inhibition of nuclear factor-kappa B (NF-κB) expression, which inhibits the growth of cancer cells and the activity of the proteasome both *in vivo* and *in vitro*. As a crucial transcription factor, NF-κB is vital for cell growth, invasion, metastasis, and angiogenesis (45). Cu2+ dramatically lowers the cancer cells’ bortezomib resistance, making its anticancer activities appear more promising. A new approach to conventional ubiquitin-proteasome inhibitor anticancer treatment is provided by copper complex-targeted UPS (46).

### 5.3 Inhibiting angiogenesis

Malignant angiogenesis encourages cancer cell growth, invasion, and metastasis since neoangiogenesis is the first stage of cancer proliferation and metastasis (47). The main component in stimulating angiogenesis is copper, which can directly promote endothelial cell migration, expansion, and the production of fibronectin (48). Hypoxia-inducible factor (HIF-1) can bind to copper and activate essential elements that control angiogenesis (49, 50). Angiogenin is a molecule that copper attaches to, stimulating endothelial cells to initiate angiogenesis (51). Copper deficiency will shut off the angiogenic switch, stop endothelial cells from proliferating, and stop the cell cycle. A novel therapeutic approach used in the treatment of cancers is copper depletion to prevent cancer angiogenesis (52). Tetrathiomolybdate (TTM), which inhibits cancer angiogenesis by reacting with copper ions to generate insoluble copper-molybdenum-sulfur clusters, is receiving much attention in cancer therapy (53, 54). TTM has shown good promise when combined with anti-cancer medications as an adjuvant. Since copper-mediated anti-angiogenic effects can block blood vessels required for cancer growth and metastasis and reshape the cancer immune microenvironment, immune checkpoint inhibitors (ICI) can promote vascular normalization, making the combination of immunotherapy with copper complexes a new target for anticancer therapy (55), which is vital in reducing cancer cell proliferation and inducing cell death (56, 57).

## 6 Cuproptosis

### 6.1 Definition of cuproptosis

Apoptosis, scorch death, autophagy, necroptosis, and metal-induced iron death are the principal types of cell death that are now understood (58, 59). Although the human body needs heavy metal ions, too little or too much can cause cell death (60). Cuproptosis, a recently identified unique form of cell death, is copper-dependent, changeable, and intricately linked to mitochondrial respiration (61). Tsvetkov et al. put out this idea. The researchers first tested 489 distinct cell types utilizing carriers for copper ions and showed that too many accumulated copper ions could result in cell death. The researchers discovered a failure to inhibit ES-induced death of non-small cell lung cancer A549 cells and NCIH2030 cells using all known inhibitors of cell death modalities after targeted knockdown of the BCL2-Associated X (BAX) and Recombinant Bcl2 Antagonist/Killer 1 (BAK1) genes. This suggests that ES-induced cancer cell death differs from known cell death mechanisms. They therefore proposed the name “cuproptosis” for this novel cell death process. Cuproptosis is a unique RCD route with a different lethal tool from oxidative stress-related cell death (62). Research shows that cuproptosis plays an important role in the occurrence and progress of many diseases, including cancer, atherosclerosis, rheumatoid arthritis, acute liver injury, novel coronavirus pneumonia, Wilson disease, Alzheimer’s disease, Parkinson’s disease etc.

### 6.2 Mechanism of cuproptosis

**Figure 2:**
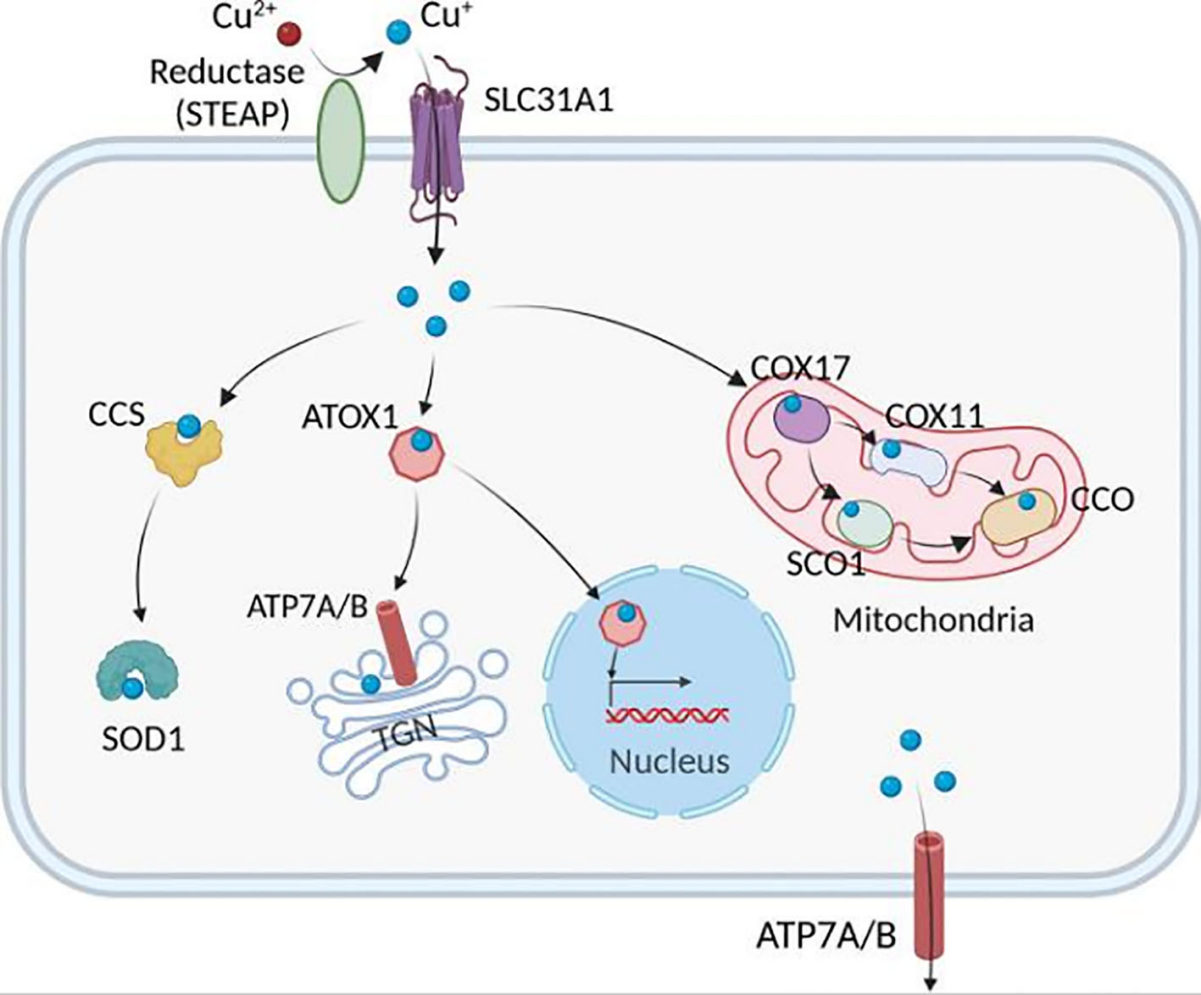
The pathways that mediate cellular Cu metabolism. Extracellular Cu2+ is reduced by the reductase STEAP to Cu+, which is transported into the cell by the Cu transporter CTR1, where it is delivered to cytosolic Cu chaperones such as CCS and SOD1 and then delivered to specific subcellular compartments such as the mitochondria, TGN, and nucleus. In the mitochondria, Cu is involved in the respiratory chain and redox pathways via binding to CCO. In the mitochondrial intermembrane space, COX17 binds to and delivers Cu to either SCO1 or COX11, which transfers Cu to the cytochrome oxidase subunit. In the nucleus, Cu can bind to transcription factors and drive gene expression. Finally, in the TGN, the Cu+-ATPase transporters ATP7A and ATP7B transfer Cu from the cytosol to the TGN lumen, where it activates Cu-dependent enzymes in the secretory pathway. ATOX1, antioxidant 1 copper chaperone; ATP7A and ATP7B, ATPase copper transporter 7A and 7B, respectively; CCO, cytochrome c oxidase; CCS, copper chaperone for superoxide dismutase; COX17, cytochrome c oxidase copper chaperone 17, COX11, cytochrome c oxidase copper chaperone 11, SCO1, synthesis of cytochrome c oxidase 1, SOD1, superoxide dismutase 1, STEAP, the six-transmembrane epithelial antigen of the prostate, SLC31A1, solute carrier family 31 members 1, TGN, trans-Golgi network (17).

Tsvetkov et al. discovered that ES-induced cell death might be connected to mitochondrial respiration. Cells relying on glycolysis are around 1000 times more sensitive to copper ion inducers than cells depending on mitochondrial respiration. Conversely, ferredoxin 1 (FDX1) lowers CU2+ to CU+, encouraging lipoylation and aggregation of enzymes responsible for controlling the mitochondrial TCA cycle (63). On the other hand, FDX1 results in the instability of iron-sulfur proteins (Fe-S), which sets off a stress response in the mitochondria and produces cuproptosis (64). In addition to copper ion carriers, copper importers and the ATP-dependent copper transporter 7A (ATP7A) (65) control the intracellular concentration of copper ions to hold the degree of cuproptosis sensitivity (66). The copper chelator GSH, which contains thiols, protects against cuproptosis. The electron transfer chain (ETC) complex inhibitor and the mitochondrial pyruvate carrier (MPC) inhibitor UK5099 prevent ES-induced cuproptosis. cuproptosis brought on by ES (**Figure 2**).

### 6.3 Copper death-related genes

Tsvetkov et al. identified ten copper death-related genes (CRGs) associated explicitly with the cuproptosis metabolic pathway (67), including seven positively regulated genes: FDX1, lipoic acid synthase (LIAS), lipoyl transferase-1 (LIPT1), dihydrolipoamide dehydrogenase (DLD), dihydrolipoamideS-acetyltransferase (DLAT), recombinant Pyruvate dehydrogenase alpha 1 (PDHA1), and pyruvate dehydrogenase (PDHB) (68–70). Metal transcription factor 1 (MTF), glutaminase (GLS), and cyclin-dependent kinase inhibitor 2A (CDKN2A) are three genes that are negatively regulated (71–73). A future study will focus on the precise function of these genes in cuproptosis and the potency of copper toxicity in cancer management (74).

As an ETC carrier, FDX, a member of the small molecular family Fe-S, is frequently utilized in various metabolic activities in living things (75). The decrease of mitochondrial cytochrome and the manufacture of different steroid hormones are both processes that FDX1, an isoform of the FDX family, aids. Cuproptosis, which converts Cu2+ to the more toxic Cu+, causes cytotoxic stress and, as a result, causes cellular cuproptosis, and FDX1 is a co-regulator of this process (76). protein Lysine can be modified post-translationally by a process known as lipoylation, and FDX1 and protein lipoylation share a family relationship (77). A considerable reduction in cellular respiration is caused by the total loss of lipoylation of the DLAT and DLST proteins when FDX1 is knocked down. These findings imply that FDX1, an essential gene in ES-induced copper-dependent cell death and an upstream regulator of protein lipoylation (78), is involved in ES-induced copper-dependent cell death. The lipoylation of mitochondrial proteins was enhanced by copper (79). The degradation of Fe-S cluster proteins by FDX1 may promote copper toxicity. Unrelated to the cuproptosis protein in cancer, FDX1 may act as an oncogene (80). Copper overload causes the aggregation of DLAT, a decrease in Fe-S cluster protein levels, an increase in the number of heat shock protein 70, and protein toxicity stress, leading to the occurrence of cuproptosis.

Three significant genes that code for the mitochondrial lipoic acid pathway are LIAS, LIPT1, and DLD (81). LIAS and LIPT1 are intricately connected to mitochondrial fatty acid production. An essential gene for mitochondrial protein lipoylation is DLAT. By acetylating 6-glucose-phosphate dehydrogenase (6PGD), DLAT increases enzyme activity, boosting nucleic acid synthesis and encouraging the growth of cancer cell lines (82). The oxidative decarboxylation of pyruvate to acetyl-CoA is made possible by the catalytic action of the pyruvate dehydrogenase complex (PDHC), which connects glycolysis, TCA, and oxidative phosphorylation. PDHA1, the PDHC’s active regulatory site The cuproptosis-related gene PDHA1 is crucial for the metabolic transformation of cancer as it regulates the cuproptosis process (83). The level of PDHA1 expression is significantly higher in cervical squamous cell carcinoma tissues than in paraneoplastic squamous epithelial tissues. It is correlated with patient age, the depth of cancer infiltration, pelvic lymph node metastasis, and International Federation of Gynecology and Obstetrics (FIGO) staging (84, 85). In hepatocellular cancer cell lines, overexpression of PDHA1 controls the activity of the TCA enzyme, prevents aerobic glycolysis, and intensifies mitochondrial-regulated apoptotic signaling pathways (86). According to specific research, CDKN2A changes in melanoma patients with CDKN2A loss may serve as a possible signal for anticipating the effectiveness of melanoma immunotherapy (87).

#### 6.3.1 CRGs regulate metabolism in cancer cells

Pathway Enrichment Analysis revealed that glutamate metabolism, TCA, and p53 signaling pathways were primarily enriched in CRGs (88). The amino acids alanine, glutamate, and aspartate all have metabolic signaling pathways connected to glucose metabolism. Metabolic reprogramming involves the p53 signaling pathway (89). TCA, glycolysis, and other processes are part of the central carbon metabolism in cancer (90). Cellular metabolism is closely tied to each of the signaling pathways mentioned above. The sensitivity of cancer cells to therapeutic drugs can be impacted by cellular metabolism, which is essential for cancer development and metastasis. Through control of cancer cell metabolism, CRGs and their related genes may influence the onset and progression of the disease (91).

#### 6.3.2 CRGs and immune cell infiltration

Analysis of immune cell infiltration revealed a strong correlation between the expression of CRGs and the quantity of various immune cells present (92). The presentation of CRGs was negatively connected with plasma cell-like follicular dendritic cells, while the expression of CRGs was favorably correlated with T helper cells. Major mitochondrial autoantigens known as DLAT-associated complexes increase CD4+ T cell and CD8+ T cell reactivity. The cancer CDKN2A was discovered. CRGs may influence cell metabolism in tumor microenvironment (TMT) by controlling several pathways, including cancer cell growth and apoptosis and the proportion of immune cells and other non-cancer cells. As a part of the TMT, immune cell infiltration may also control the growth and remission of cancers (93).

### 6.4 Cuproptosis affects mitochondrial function

Mitochondrial respiration and cuproptosis are related (94, 95). Since copper ions are primarily stored in mitochondria, an imbalance in intracellular copper metabolism can have cytotoxic effects and lead to disease onset (96). Cuproptosis may be related to altered mitochondrial activity, as evidenced by a considerable reduction in sensitivity to copper degradation when cells are placed in a hypoxic environment and forced to conduct anaerobic glycolysis (97). Both apoptosis and iron death are caused by mitochondrial stress, which can result in a severe decrease in mitochondrial membrane potential. Inhibiting glucose metabolism reduces the malignant potential of cancer cells and makes them more sensitive to copper ion carrier therapy for the treatment of cancers (98). This is because glycolysis is crucial for the proliferation of cancer cells. According to recent research, antioxidants for the mitochondria can considerably lower cuproptosis levels (99). Cuproptosis, therefore, has a high potential for use in anti-cancer therapy due to its superior anti-cancer mechanism (100).

### 6.5 Cuproptosis regulates the mechanism of cancer damage

The inflammatory response to cellular damage is impacted by cuproptosis (101). By lowering CD45 levels following cerebral ischemia injury, increasing Iba1 immunoreactivity, and changing the shape of Iba1-positive cells to modify the inflammatory response, cuproptosis can be cerebro protective. Cellular carcinogenesis depends on apoptosis (102). Burgering (103) et al. discovered that Cu, Superoxide Dismutase (SOD1) may be impacted by cuproptosis since protein kinase B (Akt) is important in regulating cell survival and death. In the bad signaling pathway, SOD1 might be necessary (104, 105). Protein lipoylation was found to be closely linked with FDX1 abundance in many cancers, and cell lines with high levels of protein lipoylation were highly susceptible to cuproptosis, indicating that copper ion carrier therapy should focus on malignancies with this metabolic profile.

## 7 Opportunities for cuproptosis cancer treatment

One of the most promising therapeutic modalities is cancer immunotherapy (106), and research on ICI, in particular, has advanced quickly (107). Current therapeutic approaches aim to selectively promote cancer cell death and prevent harming normal cells because cancer is characterized by dysregulated cell death and altered inflammatory responses (108). The need for copper is greater in cancer cells compared to normal cells. Numerous lipoylated mitochondrial proteins are expressed in high concentrations in some cancers with active respiration (109). Cu2+ in humans through bidirectional regulation has become a new target for cancer therapy since elevated copper ion levels stimulate cancer cell proliferation, metastasis, and angiogenesis (110). The fundamental component of current techniques to combat copper-induced apoptosis is using copper ion chelators and copper ion carriers to control the level of copper *in vivo* to induce apoptosis in cancer cells and produce anti-cancer effects (111). By chelating copper ions, copper ion carriers DSF and ES influence the breakdown of proteins in cancer cells, leading to copper-dependent cancer cell death (112). As a cofactor of cytochrome c oxidase, ES can control intra-mitochondrial copper ion levels, regulating cytochrome c oxidase activity and treating diseases brought on by abnormalities in human copper metabolism (113). Cytochrome C oxidase is a crucial enzyme in the mitochondrial respiratory chain.

In tumor cells, copper deficiency results in inhibition of angiogenesis, elevated ROS levels, proteasome inhibition, and mitochondrial dysfunction (114). The relationship between cuproptosis and cancer can provide new ideas for tumor salvage, and the effect of cuproptosis on cancer cells can be studied in order to reduce the copper content of cancer cells in order to mitigate the damage. When TTM is used as an antitumor drug with platinum-based chemotherapeutic drugs, the chemotherapeutic drugs become more effective and have positive synergistic effects (115). Some researchers have also discovered that Cu2+ regulates the expression of the immune checkpoint protein PD-L1 and promotes tumor immune evasion, demonstrating the potential of copper chelators as anti-tumor immune enhancers. In some cases, it is necessary to artificially increase the cellular copper content in order to alleviate inflammatory damage, and this balance must be continuously investigated in both fundamental and clinical research (116). In light of the fact that the mechanisms by which copper induces tumor cell demise vary between cancer types, their targeting and specificity must be considered in future research.

## 8 Conclusion

Copper induces tumor cell death in a variety of ways, and “cuproptosis” is a novel RCD mechanism distinct from apoptosis, iron death, autophagy, and programmed necrosis (117, 118). After a series of safety and efficacy tests, it can help translate basic chemical and biological studies of copper into potential clinical therapies and drug candidates, and it has promising applications in the field of tumor therapy. The exact mechanisms by which cuproptosis works in cancer are currently unknown, and a large number of high-quality basic studies are needed to demonstrate a causal relationship between cuproptosis and tumors. The molecular mechanisms of copper toxicity in tumors and the specific modes of evolution of cuproptosis into definitive cell death need to be further elucidated. As research continues, it is believed that more new and relevant targets and drugs will emerge to establish rational and personalized therapeutic strategies and bring new hope for the treatment of cancer patients.

## Author contributions

MW: Conceptualization, Data curation, Resources, Software, Writing – original draft, Writing – review & editing. LZ: Investigation, Software, Supervision, Validation, Writing – original draft, Writing – review & editing. SM: Conceptualization, Formal Analysis, Methodology, Project administration, Resources, Writing – original draft, Writing – review & editing. RL: Conceptualization, Data curation, Investigation, Writing – original draft, Writing – review & editing. JL: Methodology, Project administration, Supervision, Validation, Visualization, Writing – review & editing. SY: Formal Analysis, Funding acquisition, Supervision, Validation, Visualization, Writing – original draft.

## Glossary

**Table:**

RCD	Regulatory cell death
ROS	Reactive oxygen species
GSH	Glutathione
LC3	Microtubule-associated protein light chain3
DNA	deoxyribonucleic acid
PAD4	Peptidylarginine deiminase 4
ATP	Adenosine Triphosphate
DAMP	Damage-associated molecular patterns
PS	Phosphatidyl serine
TCA	tricarboxylic acid cycle
DMT1	divalent metal transporter 1
CTR1	copper transporter 1
SLC31A1	solute carrier family 31 member 1
VEGF	vascular endothelial growth factor
FGF2	fibroblast growth factor2
TNF	tumor necrosis factor
IL	interleukin
UPS	Ubiquitin proteasome system
GSSG	oxidized glutathione disulphide
ES	elesclomol
DSF	Disulfiram
DDTC	Diethyldithiocarbomate
NF-κB	nuclear factor-kappa B
HIF-1	Hypoxia-inducible factor
TTM	Tetrathiomolybdate
ICI	immune checkpoint inhibitors
BAX	BCL2-Associated X
BAK1	Recombinant Bcl2 Antagonist/Killer 1
FDX1	ferredoxin 1
Fe-S	iron-sulfur proteins
ATP7A	ATP-dependent copper transporter 7A
ETC	electron transfer chain
MPC	mitochondrial pyruvate carrier
ATOX1	antioxidant 1 copper chaperone
ATP7B	ATP-dependent copper transporter 7B
CCO	cytochrome c oxidase
CCS	copper chaperone for superoxide dismutase
COX17	cytochrome c oxidase copper chaperone 17
COX11	cytochrome c oxidase copper chaperone 11
SCO1	synthesis of cytochrome c oxidase 1
SOD1	superoxide dismutase 1
STEAP	the six-transmembrane epithelial antigen of the prostate
TGN	trans-Golgi network
CRGs	copper death-related genes
LIAS	lipoic acid synthase
LIPT1	lipoyl transferase-1
DLD	dihydrolipoamide dehydrogenase
DLAT	dihydrolipoamideS-acetyltransferase
PDHA1	ecombinant Pyruvate dehydrogenase alpha 1
PDHB	pyruvate dehydrogenase
MTF	Metal transcription factor 1
GLS	glutaminase
CDKN2A	cyclin-dependent kinase inhibitor 2A
6PGD	6-glucose-phosphate dehydrogenase
PDHC	pyruvate dehydrogenase complex
FIGO	International Federation of Gynecology and Obstetrics
TMT	tumor microenvironment
Akt	protein kinase B.

## References

[1] Bao JH Lu WC Duan H Ye YQ Li JB Liao WT. Identification of a novel cuproptosis-related gene signature and integrative analyses in patients with lower-grade gliomas. Front Immunol (2022) 13. doi: 10.3389/fimmu.2022.933973 PMC9420977 36045691

[2] Zirngibl M Assinck P Sizov A Caprariello AV Plemel JR. Oligodendrocyte death and myelin loss in the cuprizone model: an updated overview of the intrinsic and extrinsic causes of cuprizone demyelination. Mol Neurodegeneration (2022) 17(1):34. doi: 10.1186/s13024-022-00538-8 PMC9077942 35526004

[3] Huang YL Li JZ. Mechanism of copper induced tumor cell death and its research progress in tumor treatment. J Otolaryngology and Ophthalmology, Shandong University (2023), 1–23.

[4] Tsvetkov P Coy S Petrova B Dreishpoon M Verma A Abdusamad M. Copper induces cell death by targeting lipoylated TCA cycle proteins. Science (2022) 375(6586):1254–+. doi: 10.1126/science.abf0529 PMC9273333 35298263

[5] Wang Z Jin DK Zhou SS Dong NJ Ji YT An P. Regulatory roles of copper metabolism and cuproptosis in human cancers. Front Oncol (2023) 13. doi: 10.3389/fonc.2023.1123420 PMC10076572 37035162

[6] Aishajiang R Liu ZS Wang TJ Zhou L Yu D. Recent advances in cancer therapeutic copper-based nanomaterials for anticancer therapy. Molecules (2023) 28(5):2303. doi: 10.3390/molecules28052303 36903549 PMC10005215

[7] Teschke R. Aluminum, arsenic, beryllium, cadmium, chromium, cobalt, copper, iron, lead, mercury, molybdenum, nickel, platinum, thallium, titanium, vanadium, and zinc: molecular aspects in experimental liver injury. Int J Mol Sci (2022) 23(20):12213. doi: 10.3390/ijms232012213 36293069 PMC9602583

[8] Yuan HJ Xue YT Liu Y. Cuproptosis, the novel therapeutic mechanism for heart failure: a narrative review. Cardiovasc Diagnosis Ther (2022) 12(5):681–92. doi: 10.21037/cdt-22-214 PMC9622411 36329965

[9] Zhao QW Qi TG. The implications and prospect of cuproptosis-related genes and copper transporters in cancer progression. Front Oncol (2023) 13. doi: 10.3389/fonc.2023.1117164 PMC10011146 36925927

[10] Nose Y Wood LK Kim BE Prohaska JR Fry RS Spears JW. Ctr1 is an apical copper transporter in mammalian intestinal epithelial cells in vivo that is controlled at the level of protein stability. J Biol Chem (2010) 285(42):32385–92. doi: 10.1074/jbc.M110.143826 PMC2952240 20699218

[11] Xu JH Hu ZA Cao H Zhang H Luo P Zhang J. Multi-omics pan-cancer study of cuproptosis core gene FDX1 and its role in kidney renal clear cell carcinoma. Front Immunol (2022) 13. doi: 10.3389/fimmu.2022.981764 PMC9810262 36605188

[12] Chakraborty J Pakrashi S Sarbajna A Dutta M Bandyopadhyay J. Quercetin attenuates copper-induced apoptotic cell death and endoplasmic reticulum stress in SH-SY5Y cells by autophagic modulation. Biol Trace Element Res (2022) 200(12):5022–41. doi: 10.1007/s12011-022-03093-x 35149956

[13] Li YQ Huang RX Li XL Li XR Yu DD Zhang MZ. Decreased expression of pyruvate dehydrogenase A1 predicts an unfavorable prognosis in ovarian carcinoma. Am J Cancer Res (2016) 6(9):2076–87. PMC5043116 27725912

[14] Qi XC Wang J Che XY Li QL Li XW Wang QF. The potential value of cuproptosis (copper-induced cell death) in the therapy of clear cell renal cell carcinoma. Am J Cancer Res (2022) 12(8):3947–66. 10.62347/ETJH6697 PMC9442008 36119838

[15] Guthrie LM Soma S Yuan S Silva A Zulkifli M Snavely TC. Elesclomol alleviates Menkes pathology and mortality by escorting Cu to cuproenzymes in mice. Science (2020) 368(6491):620–+. doi: 10.1126/science.aaz8899 PMC7304446 32381719

[16] Agarwal P Ayton S Agrawal S Dhana K Bennett DA Barnes LL. Brain copper may protect from cognitive decline and Alzheimer's disease pathology: a community-based study. Mol Psychiatry (2022) 27(10):4307–13. doi: 10.1038/s41380-022-01802-5 PMC9764421 36195639

[17] Chen LY Min JX Wang FD. Copper homeostasis and cuproptosis in health and disease. Signal Transduction Targeted Ther (2022) 7(1):378. doi: 10.1038/s41392-022-01229-y PMC9681860 36414625

[18] Zhu XY Boulet A Buckley KM Phillips CB Gammon MG Oldfather LE. Mitochondrial copper and phosphate transporter specificity was defined early in the evolution of eukaryotes. Elife (2021) 10:e64690. doi: 10.7554/eLife.64690 33591272 PMC7924939

[19] Xie JM Yang YN Gao YB He J. Cuproptosis: mechanisms and links with cancers. Mol Cancer (2023) 22(1):46. doi: 10.1186/s12943-023-01732-y 36882769 PMC9990368

[20] Palumbo CS Schilsky ML. Clinical practice guidelines in Wilson disease. Ann Trans Med (2019) 7(Suppl 2):S65. doi: 10.21037/atm.2018.12.53 PMC6531645 31179302

[21] Shanbhag V Jasmer-McDonald K Zhu S Martin AL Gudekar N Khan A. ATP7A delivers copper to the lysyl oxidase family of enzymes and promotes cancerogenesis and metastasis. Proc Natl Acad Sci USA (2019) 116(14):6836–41. doi: 10.1073/pnas.1817473116 PMC6452744 30890638

[22] Long SC Wang Y Chen YQ Fang TS Yao YB Fu K. Pan-cancer analysis of cuproptosis regulation patterns and identification of mTOR-target responder in clear cell renal cell carcinoma. Biol Direct (2022) 17(1):28. doi: 10.1186/s13062-022-00340-y 36209249 PMC9548146

[23] Babak MV Ahn D. Modulation of intracellular copper levels as the mechanism of action of anticancer copper complexes: clinical relevance. Biomedicines (2021) 9(8):852. doi: 10.3390/biomedicines9080852 34440056 PMC8389626

[24] Borobia M Villanueva-Saz S de Arcaute MR Fernandez A Verde MT Gonzalez JM. Copper poisoning, a deadly hazard for sheep. Animals (2022) 12(18):2388. doi: 10.3390/ani12182388 36139248 PMC9495211

[25] Stelmashook EV Alexandrova OP Genrikhs EE Novikova SV Salmina AB Isaev NK. Effect of zinc and copper ions on cadmium-induced toxicity in rat cultured cortical neurons. J Trace Elements Med Biol (2022) 73:127012. doi: 10.1016/j.jtemb.2022.127012 35679765

[26] Chen XY Cai Q Liang RK Zhang DJ Liu X Zhang MY. Copper homeostasis and copper-induced cell death in the pathogenesis of cardiovascular disease and therapeutic strategies. Cell Death Dis (2023) 14(2):105. doi: 10.1038/s41419-023-05639-w 36774340 PMC9922317

[27] Jiang YC Huo ZY Qi XL Zuo TM Wu ZH. Copper-induced cancer cell death mechanisms and anticancer theragnostic applications of copper complexes. Nanomedicine (2022) 17(5):303–24. doi: 10.2217/nnm-2021-0374 35060391

[28] Cobine PA Moore SA Leary SC. Getting out what you put in Copper in mitochondria and its impacts on human disease. Biochim Et Biophys Acta-Molecular Cell Res (2021) 1868(1):118867. doi: 10.1016/j.bbamcr.2020.118867 PMC7680424 32979421

[29] Song Q Zhou R Shu FP Fu W. Cuproptosis scoring system to predict the clinical outcome and immune response in bladder cancer. Front Immunol (2022) 13. doi: 10.3389/fimmu.2022.958368 PMC9386055 35990642

[30] Chen X Kang R Kroemer G Tang DL. Broadening horizons: the role of ferroptosis in cancer. Nat Rev Clin Oncol (2021) 18(5):280–96. doi: 10.1038/s41571-020-00462-0 33514910

[31] Farhan M Rizvi A. Understanding the prooxidant action of plant polyphenols in the cellular microenvironment of Malignant cells: role of copper and therapeutic implications. Front Pharmacol (2022) 13. doi: 10.3389/fphar.2022.929853 PMC9251333 35795551

[32] Alhasawi MAI Aatif M Muteeb G Alam MW El Oirdi M Farhan M. Curcumin and its derivatives induce apoptosis in human cancer cells by mobilizing and redox cycling genomic copper ions. Molecules (2022) 27(21):7410. doi: 10.3390/molecules27217410 36364236 PMC9659251

[33] Farshori NN Siddiqui MA Al-Oqail MM Al-Sheddi ES Al-Massarani SM Ahamed M. Copper oxide nanoparticles exhibit cell death through oxidative stress responses in human airway epithelial cells: a mechanistic study. Biol Trace Element Res (2022) 200(12):5042–51. doi: 10.1007/s12011-022-03107-8 35000107

[34] Gupte A Mumper RJ. Elevated copper and oxidative stress in cancer cells as a target for cancer treatment. Cancer Treat Rev (2009) 35(1):32–46. doi: 10.1016/j.ctrv.2008.07.004 18774652

[35] Yip NC Fombon IS Liu P Brown S Kannappan V Armesilla AL. Disulfiram modulated ROS-MAPK and NFκB pathways and targeted breast cancer cells with cancer stem cell-like properties. Br J Cancer (2011) 104(10):1564–74. doi: 10.1038/bjc.2011.126 PMC3101904 21487404

[36] Yang D Xiao PY Qiu BT Yu HF Teng CB. Copper chaperone antioxidant 1: multiple roles and a potential therapeutic target. J Mol Medicine-Jmm (2023) 101(5):527–42. doi: 10.1007/s00109-023-02311-w 37017692

[37] Nagai M Vo NH Ogawa LS Chimmanamada D Inoue T Chu J. The oncology drug elesclomol selectively transports copper to the mitochondria to induce oxidative stress in cancer cells. Free Radical Biol Med (2012) 52(10):2142–50. doi: 10.1016/j.freeradbiomed.2012.03.017 22542443

[38] Li X Ma ZS Mei LH. Cuproptosis-related gene SLC31A1 is a potential predictor for diagnosis, prognosis and therapeutic response of breast cancer. Am J Cancer Res (2022) 12(8):3561–+. PMC9442001 36119835

[39] Narayanan S Cai CY Assaraf YG Guo HQ Cui QB Wei LY. Targeting the ubiquitin-proteasome pathway to overcome anti-cancer drug resistance. Drug Resistance Updates (2020) 48:100663. doi: 10.1016/j.drup.2019.100663 31785545

[40] Cobine PA Brady DC. Cuproptosis: Cellular and molecular mechanisms underlying copper-induced cell death. Mol Cell (2022) 82(10):1786–7. doi: 10.1016/j.molcel.2022.05.001 35594843

[41] Chen D Cui QZC Yang HJ Dou QP. Disulfiram, a clinically used anti-alcoholism drug and copper-binding agent, induces apoptotic cell death in breast cancer cultures and xenografts *via* inhibition of the proteasome activity. Cancer Res (2006) 66(21):10425–33. doi: 10.1158/0008-5472.CAN-06-2126 17079463

[42] Oliveri V Lanza V Milardi D Viale M Maric I Sgarlata C. Amino- and chloro-8-hydroxyquinolines and their copper complexes as proteasome inhibitors and antiproliferative agents. Metallomics (2017) 9(10):1439–46. doi: 10.1039/C7MT00156H 28932850

[43] Michalczyk K Cymbaluk-Ploska A. The role of zinc and copper in gynecological Malignancies. Nutrients (2020) 12(12):3732. doi: 10.3390/nu12123732 33287452 PMC7761859

[44] Skrott Z Mistrik M Andersen KK Friis S Majera D Gursky J. Alcohol-abuse drug disulfiram targets cancer *via* p97 segregate adaptor NPL4. Nature (2017) 552(7684):194. doi: 10.1038/nature25016 29211715 PMC5730499

[45] Han J Liu L Yue X Chang J Shi W Hua Y. A binuclear complex constituted by diethyldithiocarbamate and copper(I) functions as a proteasome activity inhibitor in pancreatic cancer cultures and xenografts. Toxicol Appl Pharmacol (2013) 273(3):477–83. doi: 10.1016/j.taap.2013.09.009 24060341

[46] Hughes RE Elliott RJR Li XD Munro AF Makda A Carter RN. Multiparametric high-content cell painting identifies copper ionophores as selective modulators of esophageal cancer phenotypes. ACS Chem Biol (2022) 17(7):1876–89. doi: 10.1021/acschembio.2c00301 PMC9295120 35696676

[47] Yin R Wang H Li C Wang L Lai S Yang X. Induction of apoptosis and autosis in cardiomyocytes by the combination of homocysteine and copper *via* NOX-mediated p62 expression. Cell Death discovery (2022) 8(1):75. doi: 10.1038/s41420-022-00870-4 35190552 PMC8860999

[48] Caro-Ramírez JY Rivas MG Gonzalez PJ Williams PAM Naso LG Ferrer EG. Copper(II) cation and bathophenanthroline coordination enhance the therapeutic effects of naringenin against lung cancer cells. BioMetals (2022) 35(5):1059–76. doi: 10.1007/s10534-022-00422-4 35931942

[49] Agnieszka Z Maciej K. Hypoxia-inducible factor-1 in physiological and pathophysiological angiogenesis: applications and therapies. BioMed Res Int (2015) 2015:549412. doi: 10.1155/2015/549412 26146622 PMC4471260

[50] Wang J Luo C Shan CL You QC Lu JY Elf SN. Inhibition of human copper trafficking by a small molecule significantly attenuates cancer cell proliferation. Nat Chem (2015) 7(12):968–79. doi: 10.1038/nchem.2381 PMC4725056 26587712

[51] Chi H Peng GG Wang R Yang FY Xie XX Zhang JH. Cuproptosis programmed-cell-death-related lncRNA signature predicts prognosis and immune landscape in PAAD patients. Cells (2022) 11(21):3436. doi: 10.3390/cells11213436 36359832 PMC9658590

[52] Ge EJ Bush AI Casini A Cobine PA Cross JR DeNicola GM. Connecting copper and cancer: from transition metal signalling to metaplasia. Nat Rev Cancer (2022) 22(2):102–13. doi: 10.1038/s41568-021-00417-2 PMC8810673 34764459

[53] Brewer GJ. Copper-lowering therapy with tetrathiomolybdate for cancer and diseases of fibrosis and inflammation. J Trace Elements Exp Med (2003) 16(4):191–9. doi: 10.1002/jtra.10045

[54] Czlonkowska A Litwin T Dusek P Ferenci P Lutsenko S Medici V. Wilson disease. Nat Rev Dis Primers (2018) 4(1):21. doi: 10.1038/s41572-018-0018-3 30190489 PMC6416051

[55] Morisawa A Okui T Shimo T Ibaragi S Okusha Y Ono M. Ammonium tetrathiomolybdate enhances the anticancer effects of cetuximab *via* the suppression of osteoclastogenesis in head and neck squamous carcinoma. Int J Oncol (2018) 52(3):989–99. doi: 10.3892/ijo.2018.4242 29328370

[56] Lan QH Du CC Yu RJ Zhai JY Shi YN Kou LF. Disulfiram-loaded copper sulfide nanoparticles for potential anti-glioma therapy. Int J Pharmaceutics (2021) 607 :120978. doi: 10.1016/j.ijpharm.2021.120978 34371152

[57] Cao HZ Yang WT Zheng PS. Cytotoxic effect of disulfiram/copper on human cervical cancer cell lines and LGR5-positive cancer stem-like cells. BMC Cancer (2022) 22(1):1–13. doi: 10.1186/s12885-022-09574-5 35534815 PMC9082913

[58] Zhang SP Xin W Anderson GJ Li RB Gao L Chen SG. Double-edge sword roles of iron in driving energy production versus instigating ferroptosis. Cell Death Dis (2022) 13(1):40. doi: 10.1038/s41419-021-04490-1 35013137 PMC8748693

[59] Bock FJ Tait SWG. Mitochondria as multifaceted regulators of cell death. Nat Rev Mol Cell Biol (2020) 21(2):85–100. doi: 10.1038/s41580-019-0173-8 31636403

[60] Tong XH Tang R Xiao MM Xu J Wang W Zhang B. Targeting cell death pathways for cancer therapy: recent developments in necroptosis, pyroptosis, ferroptosis, and cuproptosis research. J Hematol Oncol (2022) 15(1):174. doi: 10.1186/s13045-022-01392-3 36482419 PMC9733270

[61] Litwin T Bembenek J Antos A Kurkowska-Jastrzebska I Przybylkowski A Skowronska M. The maternal and fetal outcomes of pregnancy in wilson's disease: A systematic literature review and meta-analysis. Biomedicines (2022) 10(9):2072. doi: 10.3390/biomedicines10092072 36140172 PMC9495510

[62] Cui XN Wang Y Liu H Shi MJ Wang JW Wang YF. The molecular mechanisms of defective copper metabolism in diabetic cardiomyopathy. Oxid Med Cell Longevity (2022) 2022:5418376. doi: 10.1155/2022/5418376 PMC9553361 36238639

[63] Zeng R Peng B Peng EM. Downregulated copper homeostasis-related gene FOXO1 as a novel indicator for the prognosis and immune response of breast cancer. J Immunol Res (2022) 2022:9140461. doi: 10.1155/2022/9140461 35800988 PMC9256448

[64] Wang L Cao Y Guo W Xu J. High expression of cuproptosis-related gene FDX1 in relation to good prognosis and immune cells infiltration in colon adenocarcinoma (COAD). J Cancer Res Clin Oncol (2023) 149(1):15–24. doi: 10.1007/s00432-022-04382-7 36173462 PMC9889456

[65] da Silva DA De Luca A Squitti R Rongioletti M Rossi L MaChado CML. Copper in cancers and the use of copper-based compounds in cancer treatment. J Inorganic Biochem (2022) 226:111634. doi: 10.1016/j.jinorgbio.2021.111634 34740035

[66] Qasem Z Pavlin M Ritacco I Avivi MY Meron S Hirsch M. Disrupting Cu trafficking as a potential therapy for cancer. Front Mol Biosci (2022) 9. doi: 10.3389/fmolb.2022.1011294 PMC9589254 36299299

[67] Duan WJ He RR. Cuproptosis: copper-induced regulated cell death. Sci China-Life Sci (2022) 65(8):1680–2. doi: 10.1007/s11427-022-2106-6 35925445

[68] Dorsam B Fahrer J. The disulfide compound α-lipoic acid and its derivatives: A novel class of anticancer agents targeting mitochondria. Cancer Letters (2016) 371(1):12–9. doi: 10.1016/j.canlet.2015.11.019 26604131

[69] Sha SN Si LY Wu XR Chen YB Xiong H Xu Y. Prognostic analysis of cuproptosis-related gene in triple-negative breast cancer. Front Immunol (2022) 13. doi: 10.3389/fimmu.2022.922780 PMC9376234 35979353

[70] Liu HR. Pan-cancer profiles of the cuproptosis gene set. Am J Cancer Res (2022) 12(8):4074–+. doi: 10.3389/fonc.2022.952290 PMC9442004 36119826

[71] Sharma P Goyal D Chudasama B. Antibacterial Activity of Colloidal Copper Nanoparticles against Gram-negative (Escherichia coli and Proteus vulgaris) Bacteria. Lett Appl Microbiol (2022) 74(5):695–706. doi: 10.1111/lam.13655 35034356

[72] Rayess H Wang MB Srivatsan ES. Cellular senescence and cancer suppressor gene p16. Int J Cancer (2012) 130(8):1715–25. doi: 10.1002/ijc.27316 PMC3288293 22025288

[73] Cen D Brayton D Shahandeh B Meyskens Jr Farmer FL PJ. Disulfiram facilitates intracellular cu uptake and induces apoptosis in human melanoma cells. J Medicinal Chem (2004) 47(27):6914–20. doi: 10.1021/jm049568z 15615540

[74] Jiang ZT Sha GY Zhang WJ Zhang ZL Liu T Wang DR. The vast potential of targeting copper status in the treatment of colorectal cancer. Clin Trans Oncol (2023) 25(7):1977–90. doi: 10.1007/s12094-023-03107-7 36781599

[75] Baszuk P Marciniak W Derkacz R Jakubowska A Cybulski C Gronwald J. Blood copper levels and the occurrence of colorectal cancer in Poland. Biomedicines (2021) 9(11):1628. doi: 10.3390/biomedicines9111628 34829856 PMC8615693

[76] Hu Q Wang RT Ma HY Zhang ZW Xue Q. Cuproptosis predicts the risk and clinical outcomes of lung adenocarcinoma. Front Oncol (2022) 12. doi: 10.3389/fonc.2022.922332 PMC9393616 36003780

[77] Wang T Liu YF Li Q Luo Y Liu DW Li B. Cuproptosis-related gene FDX1 expression correlates with the prognosis and cancer immune microenvironment in clear cell renal cell carcinoma. Front Immunol (2022) 13. doi: 10.3389/fimmu.2022.999823 PMC9549781 36225932

[78] Diz M Duran-Carril ML Castro J Alvo S Bada L Vina D. Anticancer activity of copper(II) complexes with Schiff bases derived from N' -tosyl benzene-1,2-diamine. J Inorganic Biochem (2022) 236:111975. doi: 10.1016/j.jinorgbio.2022.111975 36055108

[79] Jiapaer Z Zhang L Ma W Liu H Li C Huang W. Disulfiram-loaded hollow copper sulfide nanoparticles show anti-cancer effects in preclinical models of colorectal cancer. Biochem Biophys Res Commun (2022) 635:291–8. doi: 10.1016/j.bbrc.2022.10.027 36327916

[80] Yun YH Wang Y Yang ED Jing X. Cuproptosis-related gene-SLC31A1, FDX1 and ATP7B-polymorphisms are associated with risk of lung cancer. Pharmacogenomics Personalized Med (2022) 15:733–42. doi: 10.2147/PGPM.S372824 PMC9342429 35923305

[81] Tang DL Chen X Kroemer G. Cuproptosis: a copper-triggered modality of mitochondrial cell death. Cell Res (2022) 32(5):417–8. doi: 10.1038/s41422-022-00653-7 PMC9061796 35354936

[82] Davis CI Gu XX Kiefer RM Ralle M Gade TP Brady DC. Altered copper homeostasis underlies the sensitivity of hepatocellular carcinoma to copper chelation. Metallomics (2020) 12(12):1995–2008. doi: 10.1039/d0mt00156b 33146201 PMC8315290

[83] Li J Wu F Li CF Sun SY Feng C Wu HZ. The cuproptosis-related signature predicts prognosis and indicates the immune microenvironment in breast cancer. Front Genet (2022) 13. doi: 10.3389/fgene.2022.977322 PMC9548612 36226193

[84] Lei L Tan L Sui L. A novel cuproptosis-related gene signature for predicting prognosis in cervical cancer. Front Genet (2022) 13. doi: 10.3389/fgene.2022.957744 PMC9453033 36092887

[85] Zhang M Shi M Zhao Y. Association between serum copper levels and cervical cancer risk: a meta-analysis. Bioscience Rep (2018) 38(4):BSR20180161. doi: 10.1042/BSR20180161 PMC6435553 29519960

[86] Wang YX Zhang YF Wang LR Zhang N Xu WQ Zhou JM. Development and experimental verification of a prognosis model for cuproptosis-related subtypes in HCC. Hepatol Int (2022) 16(6):1435–47. doi: 10.1007/s12072-022-10381-0 36065073

[87] Lv HZ Liu X Zeng XH Liu YT Zhang CJ Zhang Q. Comprehensive analysis of cuproptosis-related genes in immune infiltration and prognosis in melanoma. Front Pharmacol (2022) 13. doi: 10.3389/fphar.2022.930041 PMC9273972 35837286

[88] Yang LF Zhang YW Wang Y Jiang P Liu FP Feng NH. Ferredoxin 1 is a cuproptosis-key gene responsible for cancer immunity and drug sensitivity: A pan-cancer analysis. Front Pharmacol (2022) 13. doi: 10.3389/fphar.2022.938134 PMC9532935 36210836

[89] Xiong C Ling H Hao Q Zhou X. Cuproptosis: p53-regulated metabolic cell death? Cell Death Differ (2023) 30(4):876–84. doi: 10.1038/s41418-023-01125-0 PMC10070433 36755067

[90] Peng XY Zhu JF Liu SC Luo C Wu X Liu ZT. Signature construction and molecular subtype identification based on cuproptosis-related genes to predict the prognosis and immune activity of patients with hepatocellular carcinoma. Front Immunol (2022) 13. doi: 10.3389/fimmu.2022.990790 PMC9555242 36248822

[91] Zhang Z Zeng XY Wu YH Liu Y Zhang X Song ZW. Cuproptosis-related risk score predicts prognosis and characterizes the cancer microenvironment in hepatocellular carcinoma. Front Immunol (2022) 13. doi: 10.3389/fimmu.2022.925618 PMC9311491 35898502

[92] Ding FX Li F Tang DS Wang B Liu JY Mao XY. Restoration of the immunogenicity of cancer cells for enhanced cancer therapy *via* nanoparticle-mediated copper chaperone inhibition. Angewandte Chemie-International Edition (2022) 61(31):e202203546. doi: 10.1002/anie.202203546 35642869

[93] Engin AB Engin ED Engin A. Can iron, zinc, copper and selenium status be a prognostic determinant in COVID-19 patients? Environ Toxicol Pharmacol (2022) 95:14. doi: 10.1016/j.etap.2022.103937 PMC9307469 35882309

[94] Bian ZL Fan R Xie LM. A novel cuproptosis-related prognostic gene signature and validation of differential expression in clear cell renal cell carcinoma. Genes (2022) 13(5):851. doi: 10.3390/genes13050851 35627236 PMC9141858

[95] Zhao JA Guo SC Schrodi SJ He DY. Cuproptosis and cuproptosis-related genes in rheumatoid arthritis: Implication, prospects, and perspectives. Front Immunol (2022) 13. doi: 10.3389/fimmu.2022.930278 PMC9386151 35990673

[96] Coelho FC Cerchiaro G Santo Araujo SE Daher JPL Cardoso SA Coelho GF. Is there a connection between the metabolism of copper, sulfur, and molybdenum in alzheimer's disease? New insights on disease etiology. Int J Mol Sci (2022) 23(14):7935. doi: 10.3390/ijms23147935 35887282 PMC9324259

[97] Malik M Switlicka A Bienko A Komarnicka UK Bienko DC Koziel S. Copper(II) complexes with 2-ethyl pyridine and related hydroxyl pyridine derivatives: structural, spectroscopic, magnetic and anticancer in *vitro* studies. RSC Advances (2022) 12(42):27648–65. doi: 10.1039/D2RA05133H PMC9516696 36276031

[98] Shan JS Geng R Zhang Y Wei JT Liu JH Bai JL. Identification of cuproptosis-related subtypes, the establishment of a prognostic model and cancer immune landscape in endometrial carcinoma. Comput Biol Med (2022) 149:105988. doi: 10.1016/j.compbiomed.2022.105988 36007289

[99] Liu J Liu Y Wang Y Kang R Tang DL. HMGB1 is a mediator of cuproptosis-related sterile inflammation. Front Cell Dev Biol (2022) 10. doi: 10.3389/fcell.2022.996307 PMC9534480 36211458

[100] Feng AQ He LN Chen T Xu MD. A novel cuproptosis-related lncRNA nomogram to improve the prognosis prediction of gastric cancer. Front Oncol (2022) 12. doi: 10.3389/fonc.2022.957966 PMC9465020 36106123

[101] Kahlson MA Dixon SJ. Copper-induced cell death. Sci (New York NY) (2022) 375(6586):1231–2. doi: 10.1126/science.abo3959 35298241

[102] Li GX Peng LH Wu MJ Zhao YP Cheng Z Li G. Appropriate level of cuproptosis may be involved in alleviating pulmonary fibrosis. Front Immunol (2022) 13. doi: 10.3389/fimmu.2022.1039510 PMC9806118 36601107

[103] Burgering BM Coffer PJ. Protein kinase B (c-Akt) in phosphatidylinositol-3-OH kinase signal transduction. Nature (1995) 376(6541):599–602. doi: 10.1038/376599a0 7637810

[104] McAlary L Shephard VK Wright GSA Yerbury JJ. A copper chaperone-mimetic polytherapy for SOD1-associated amyotrophic lateral sclerosis. J Biol Chem (2022) 298(3):101612. doi: 10.1016/j.jbc.2022.101612 35065969 PMC8885447

[105] Saito A Hayashi T Okuno S Ferranddrake M Chan PH. Overexpression of Copper/Zinc Superoxide Dismutase in Transgenic Mice Protects against Neuronal Cell Death after Transient Focal Ischemia by Blocking Activation of the Bad Cell Death Signaling Pathway. J Neurosci Off J Soc Neurosci (2003) 23(5):1710–8. doi: 10.1523/JNEUROSCI.23-05-01710.2003 PMC6741974 12629175

[106] Bao XZ Wang Q Ren XR Dai F Zhou B. A hydrogen peroxide-activated Cu(II) pro-ionophore strategy for modifying naphthazarin as a promising anticancer agent with high selectivity for generating ROS in HepG2 cells over in L02 cells. Free Radical Biol Med (2020) 152:597–608. doi: 10.1016/j.freeradbiomed.2019.12.001 31805398

[107] Liu JS Lu YY Dai YY Shen Y Zeng C Liu XL. A comprehensive analysis and validation of cuproptosis-associated genes across cancers: Overall survival, the cancer microenvironment, stemness scores, and drug sensitivity. Front Genet (2022) 13. doi: 10.3389/fgene.2022.939956 PMC9465292 36105090

[108] Xia SY Jia HX Qian ZP Xiu YC. Role of copper ionophore-induced death in the immune microenvironment and clinical prognosis of ccRCC: An integrated analysis. Front Genet (2022) 13. doi: 10.3389/fgene.2022.994999 PMC9574041 36263424

[109] Oliveri V. Selective targeting of cancer cells by copper ionophores: an overview. Front Mol Biosci (2022) 9. doi: 10.3389/fmolb.2022.841814 PMC8931543 35309510

[110] Tsymbal S Li G Agadzhanian N Sun YH Zhang JZN Dukhinova M. Recent advances in copper-based organic complexes and nanoparticles for cancer theranostics. Molecules (2022) 27(20):7066. doi: 10.3390/molecules27207066 36296659 PMC9611640

[111] Tsymbal SA Refeld AG Kuchur OA. The p53 Cancer Suppressor and Copper Metabolism: An Unrevealed but Important Link. Mol Biol (2022) 56(6):979–92. doi: 10.1134/S0026893322060188 36475489

[112] Tsvetkov P Detappe A Cai K Keys HR Brune Z Ying WW. Mitochondrial metabolism promotes adaptation to proteotoxic stress. Nat Chem Biol (2019) 15(7):681–+. doi: 10.1038/s41589-019-0291-9 PMC8183600 31133756

[113] Tsang T Posimo JM Gudiel AA Cicchini M Brady DC. Copper is an essential regulator of the autophagic kinases ULK1/2 to drive lung adenocarcinoma. Nat Cell Biol (2019) 22(4):412–24. doi: 10.1038/s41556-020-0481-4 PMC7610258 32203415

[114] Farhan M Rizvi A Ali F Ahmad A Aatif M Malik A. Pomegranate juice anthocyanidins induce cell death in human cancer cells by mobilizing intracellular copper ions and producing reactive oxygen species. Front Oncol (2022) 12. doi: 10.3389/fonc.2022.998346 PMC9487716 36147917

[115] Gao XX Huang HC Pan CX Mei ZB Yin SY Zhou L. Disulfiram/copper induces immunogenic cell death and enhances CD47 blockade in hepatocellular carcinoma. Cancers (2022) 14(19):4715. doi: 10.3390/cancers14194715 36230638 PMC9564202

[116] Agarwal A Khandelwal A Pal K Khare NK Jadhav V Gurjar M. A novel pro-oxidant combination of resveratrol and copper reduce transplant-related toxicities in patients receiving high dose melphalan for multiple myeloma (RESCU 001). PloS One (2022) 17(2):e0262212. doi: 10.1371/journal.pone.0262212 35120140 PMC8815866

[117] Aschner M Skalny AV Martins AC Sinitskii AI Farina M Lu RZ. Ferroptosis as a mechanism of non-ferrous metal toxicity. Arch Toxicology (2022) 96(9):2391–417. doi: 10.1007/s00204-022-03317-y 35727353

[118] Fan YZ Cheng ZL Mao LJ Xu G Li N Zhang ML. PINK1/TAX1BP1-directed mitophagy attenuates vascular endothelial injury induced by copper oxide nanoparticles. J Nanobiotechnology (2022) 20(1):149. doi: 10.1186/s12951-022-01338-4 35305662 PMC8934125

